# Prevalence of the five newborn screening tests

**DOI:** 10.1371/journal.pone.0257282

**Published:** 2021-09-13

**Authors:** Josilene Maria Ferreira Pinheiro, Taiana Brito Menêzes Flor, Cristiane da Silva Ramos Marinho, Vanessa Cristina da Costa Pires, Luana Isabelly Carneiro de Oliveira, Mara Rúbia de Oliveira Bezerra, Jéssica Rodrigues Clementino, Fábia Barbosa de Andrade

**Affiliations:** 1 Graduate Degree in Public Health, Federal University of Rio Grande do Norte, Natal, Rio Grande do Norte, Brazil; 2 Undergraduate Student from the Nutrition Course, Federal University of Rio Grande do Norte, Natal, Rio Grande do Norte, Brazil; 3 Onofre Lopes University Hospital, Natal, Rio Grande do Norte, Brazil; Meyer Children’s University Hospital - University of Florence, ITALY

## Abstract

Neonatal screening is essential for child health and has the following purposes: (1) pulse oximetry screening to evaluate congenital heart diseases; (2) red reflex examination to investigate eye diseases; (3) newborn hearing screening test to evaluate congenital hearing diseases; (4) tongue test to evaluate the lingual frenulum and identify communication and feeding problems; (5) the Guthrie test to screen for metabolic diseases. This study investigated the prevalence of the five neonatal screening tests and its associated institutional and socio-cultural factors using a cross-sectional study with 415 mother and baby binomials from public maternity hospitals in Natal, RN, Brazil in 2019. Pearson’s chi-squared, Mann-Whitney and Poisson regression tests were used, with a significance of p ≤ 0.05 and a 95% confidence interval. The sample loss was 71 mothers (17%). The prevalence in the first week and at the end of 28 days was 93% and 99.5% (pulse oximetry screening), 60% and 97.6% (red reflex examination), 71.9% and 93.6% (Guthrie test), 35.5% and 68.2% (hearing screening test), and 19% and 48.9% (tongue test). Only 152 newborns (36.6%) underwent all five tests. The performance of the tests was associated in the final model (p ≤ 0.05) with the residence of the mothers in the state capital (PR = 1.36; 95% CI = 1.18–1.56) and the provision of guidance for mothers about the five tests in maternity hospitals (PR = 1.30; 95% CI = 1.08–1.67). None of the tests met full coverage, and regional inequities were identified indicating the need to restructure the institutions, training and qualification procedures to improve of the work processes and longitudinal care.

## Introduction

Early diagnosis, adequate treatment and medical follow-up for some diseases can prevent death and disabilities and provide a better quality of life for newborns. Neonatal screening (NS) has been part of many countries’ public policy, since the 1960, with variations in its breadth and coverage. The current front-runners are the United States and some European, Asian, and Latin American countries [1,2].

The NS in Brazil has been expanded to universal neonatal screening (*TNU* per its Portuguese acronym). It is linked to the National Policy on Children’s Health (PNAISC, per its Portuguese acronym) as one of the strategic actions of the axis of humanized and qualified newborn care. TNU has been integrated with primary health care and is part of the following health care networks throughout Brazil: The stork Network, Care Network for People with Chronic Illness and Care Network for People with Disabilities. *TNU* has been implemented in all federal units and derives from articulated actions in the three governmental spheres (federal, state, and municipal), focusing on long-term, integrated care through the early identification, follow-up, and treatment of disease [3].

Brazil’s Unified Health System (*SUS*, per its Portuguese acronym) recommends 100% coverage for the five NS tests: pulse oximetry screening, red reflex, hearing screening test, tongue test and Guthrie test. The first test practiced in Brazil was the heel prick (Guthrie test), first used in 1976 to identify phenylketonuria. However, *TNU* did not become a national standard until June 6, 2001, when Ordinance 822 created the National Neonatal Screening Program (*PNTN*, per its Portuguese acronym) for all federal states. Based on established criteria, the *PNTN* also increased the number of metabolic, genetic, enzymatic, and endocrinological diseases diagnosed by biological NS. Today, neonates are screened for six diseases (i.e. phenylketonuria, congenital hypothyroidism, sickle cell disease and other hemoglobinopathies, cystic fibrosis, congenital adrenal hyperplasia, and biotinidase deficiency); however, the *PNTN* still needs to be strengthened by screening for the 50 or so common diseases types identifiable by modern biological tests. [2,4]. The *PNTN* currently recommends blood collection between the newborns’ third and fifth day of life. Early diagnosis allows therapeutic access which can foresee severe complications.

Parts of the *PNTN* also made other tests mandatory within newborns’ first 48 hours of life. Law No. 12,303 (August 2010) required the newborn hearing screening test for evoked otoacoustic emissions. Ordinance No. 20 (June 10, 2014) required the pulse oximetry screening test to identify congenital heart diseases. Law No. 4,090 (December 16, 2015) required the red reflex examination to identify eye disorders (i.e. cataracts, congenital glaucoma, among others). Law No. 13,002 (June 20, 2014) required the tongue test to assess the lingual frenulum and identify ankyloglossia or “tongue-tie,” an anomaly which results in limitations during latch‐on and suction that can compromise breastfeeding [3,5–7].

Despite the *PNTN’s* enhancements to neonatal healthcare mandatory universal neonatal screening, multidisciplinary teams expansion in maternity hospitals and professional qualification requirements Brazil still has not achieved 100% NS. The innumerable difficulties and limitations include economic, administrative, structural, organizational, personnel training and social barriers. In turn, these obstacles mean that many of the tests which are not or cannot be performed in the maternity hospital are later conducted by primary healthcare practitioners [8,9].

The literature suggests that social and regional inequalities significantly influence the usage rate of the Guthrie test, red reflex examination, and hearing screening test, with a higher prevalence in the South and Southeast regions of Brazil and for newborns of mothers with higher education and family income levels [10–12]. However, data on factors affecting the prevalence of pulse oximetry screening and the tongue test, remain scarce and require more studies.

Therefore, this study investigated the prevalence of the five screening tests performance on newborn delivered in public maternity hospitals in Natal (Rio Grande do Norte-RN state, Brazil) during the first 28 days of life, and its associated institutional and socio-cultural factors.

## Methods

### Study design

This cross-sectional study follows the cohort study “Child Care in the Neonatal Period,” conducted from February to August 2019 to evaluate the healthcare actions recommended by the Brazilian Ministry of Health for newborns in the neonatal period.

### Study population and sample

The study population comprised mothers and newborns from the four public maternity hospitals of the city of Natal, Brazil. We anonymized the maternity hospitals, using the letters A, B, C, and D. The administrations included all three types: municipal administration (B and D), state (C) and federal (A). The last two assisted in high-risk births and specialized in high-risk pregnancy care.

The sample calculation was based on data from the Neonatal Call/2010 survey [13], which showed an approximate prevalence of 70% for care actions. The “Neonatal Call” evaluated prenatal care, maternal and child morbidity, and the breastfeeding of children under one year in the North and Northeast regions of Brazil during a multivaccination campaign conducted on June 12, 2010, in the 252 municipalities of 17 federal units that are signatories of the Pact to Reduce Infant Mortality [13].

A sample error of 5% was used for a population of 14,025 live births in 2018, a confidence level of 95% and a nonresponse rate of 24% for a total sample size of 415 mother-son pairs. The total was stratified proportionally according to the number of live births in the four public maternity hospitals: 31% in hospital A (129 mother-child pairs), 25% in hospital B (102 mother-child pairs), 23% in hospital C (96 mother-child pairs), and 21% in hospital D (88 mother-child pairs).

#### Inclusion criteria and data collection

We followed these inclusion criteria: newborn at term (≥ 37 weeks), birth weight ≥ 2500g, Apgar at 10 and 50 minutes ≥ 7, and single pregnancy. Twins, infants with congenital malformations, infants who were referred to intensive care units, and mothers who were not in good enough health to respond to the research form were excluded from the study.

The data collection was carried out daily in each maternity hospital, in the morning and afternoon shifts, for approximately two months until the expected total sample was complete. We trained the interviewers on the study organization, the instruments to be used and their application before the data collection commenced to minimize systematic and random errors. We had previously tested the collection instrument (electronic form on a touchscreen) through a pilot study with thirty mother-child binomial. After the mothers of the newborns signed the Informed Consent form (teenage mothers signed the Informed Consent Term), we proceeded to collect data on three occasions: after the newborn’s first 48 hours of life in the rooming-in unit (face-to-face interview and data collection from physical records), after the first week of life, by voice-only telephone, and at 28 days of life by voice-only telephone, with a maximum period of two days for follow-up, also by telephone call. Loss of follow-up was assumed after three unsuccessful attempts to reach a mother by telephone.

### Study variables

The study outcome was the completion frequency of all five NS tests in the newborns’ first 28 days of life: the pulse oximetry screening, red reflex examination, hearing screening test, tongue test and Guthrie test.

We chose the independent variables based on newborn care, social and health determinants, selecting the following: the administrative nature of the maternity hospital (municipal, state or federal); care complexity level (low or high); the ratio of the number of professionals per obstetric beds in the rooming-in unit (team: pediatrician, obstetrician, nurse, nursing technicians, nutritionist, speech therapist, social worker, psychologist, physical therapist and ophthalmologist); the provision of guidance on screening tests and advice to seek a health service after hospital discharge (yes or no); age (≤ 20 years, 20–29 years, > 30 years), education level (elementary school, high school or higher education); income (in Brazilian Reais) and categories (>1 minimum salary or ≤1 minimum salary); “*bolsa familia*” beneficiary family grant (yes or no); marital status (married or stable relationship; single, widow or divorced); mothers’ residence (state capital or countryside); type of delivery (vaginal or cesarean section); perception of newborns’ risk in the neonatal period (considering the risk period: yes or no); newborn physical measurements (gestational age in weeks, weight in kilograms and length and head circumference in centimeters).

### Statistical analysis

The data were tabulated using Microsoft Excel and analyzed using the IBM SPSS Statistics for Windows, Version 20.0 (Armonk, NY: IBM Corp). We initially performed a descriptive analysis of the prevalence of performing universal NS tests, presenting the results in absolute and relative frequencies. Next, we used Pearson’s chi-squared test and the Mann-Whitney test in the bivariate analysis between the exposure and outcome variables, considering the non-normal distribution of the quantitative data. We also used Poisson regression for multiple analysis and the identification of the adjusted prevalence ratio. Variables with individual characteristics with *p*≤ 0.20 were initially included and then variables with *p*< 0.10 in subsequent analyses. Variables with a *p*≤ 0.05 and a 95% confidence interval remained in the final model, meeting the established assumptions (goodness of fit, omnibus test, dispersion).

### Ethics

This study was approved by the Research Ethics Committee of the Onofre Lopes University Hospital under Opinion 3.133217 and is in line with Resolution 466/2012, which includes guidelines and regulatory standards for research involving human beings. The women who met the inclusion criteria after the selection step were consulted about their interest in participating in this research. They signed the Free and Informed Consent Form when they agreed to participate. Consent was obtained from parents or legal guardians if the participante was under 18 years old.

## Results

A total of 415 mother/newborn pairs participated in the first stage of the study, of which 45.8% came from municipal hospitals, 23.1% from state hospitals and 31.1% from federal hospitals. A total of 344 mothers (82.9%) returned our telephone calls at the end of the neonatal study period.

As shown in Table 1, the completion frequency for the universal NS tests varied by types and period. Pulse oximetry screening had the highest frequency at the end of the neonatal period, followed by the red reflex examination, the Guthrie test, the hearing screening test, and the tongue test. When the total number of tests was evaluated, all five tests were performed on only 152 (36.6%) newborns; four tests were performed on 76 (18.3%) newborns; three tests were performed on 121 (29.2%) newborns; two tests were performed on 48 (11.6%) newborns; and only one test was performed on 18 (4.3%) newborns.

**Table 1:** Completion frequency of neonatal universal screening tests (first 28 days of life) in Natal, Brazil, 2019 (n = 415).

Neonatal screening tests	First week of life			First 28 days of life		
	*n*	%	CI	*n*	%	CI
**Oximetry screening** *						
Yes	386	93.0	90.6–95.5	412	99.5	98.5–100.0
No	29	7.0	4.5–9.4	2	0.5	0.2–1.0
**Red reflex screening** *						
Yes	249	60.0	55.3–67.4	373	97.6	96.2–99.1
No	166	40.0	35.3–44.7	9	2.4	0.9–3.8
**Guthrie test** ţ						
Yes	264	63.6	59.0–68.2	350	93.6	91.2–95.9
No	151	28.1	31.8–41.0	24	6.4	4.1–8.8
**Hearing screening** *						
Yes	139	35.5	28.9–38.0	245	68.2	63.8–72.7
No	276	66.5	61.9–71.0	114	31.8	27.3–36.2
**Tongue test** *						
Yes	79	19.0	15.2–22.8	173	48.9	44.1–53.7
No	336	81.0	77.2–94.7	181	51.1	46.3–55.9

Notes

*Interview in the first 48 hours.

ţ Interview between third and seventh day of life. CI: Confidence interval.

Less than half of the participants were aged 20 to 29 years (Table 2); more than half were married or living in a stable relationship, had high school or higher education, and had a family income less than or equal to one minimum monthly salary.

**Table 2:** Association of care and maternal characteristics with the completion of the five NS tests Natal/Brazil, 2019 (n = 152).

	Total (415)	Neonatal screening tests							
				Not-adjusted			Adjusted		
	*n*	*n*	%	*P value*	PR	CI	PR	CI	*P value*
**Care characteristics**									
*Test guidance*				0.006					**0.006**
Yes	120	60	50.0		1.30	1.08–1.57	1.35	1.17–1.55	
No	295	92	31.2						
*Guidance for seeking HS*				0.100					
Yes	282	115	40.8		1.16	0.97–1.40	-	-	-
No	71	22	31.0						
**Maternal characteristics**									
*Maternal age*									
≤ 20 years	79	27	34.2	-	-	-	-	-	-
20 to 29 years	193	68	35.2						
>30 years	143	57	39.9						
*Marital status*				0.663					
Married or stable union	328	122	37.2		1.04	0.87–1.24	-	-	-
Single/divorced/widowed	87	30	34.5						
*Education level*				0.004					
High school or higher	271	112	41.3		1.23	1.07–1.42	-	-	-
Elementary school	144	40	27.8						
*Mother’s* Residence				<0.001					**<0.001**
State capital	252	112	44.4		1.36	1.18–1.56	1.29	1.07–1.56	
Countryside	163	40	24.5						
*Family income*				0.061					
>1 minimum salary	147	63	42.9		1.17	0.99–1.38	-	-	-
≤ 1 minimum salary	268	89	33.2						
*Beneficiary Family Grant*									
No	169	101	41.1	0.021	1.10	1.03–1.37	-	-	-
Yes	246	51	30.2						
*Parity*									
Multiparous	261	49	31.8	0.110	1.13	0.97–1.30	-	-	-
Primiparous	154	103	39.5						
*Delivery type *				-	-	-	-	-	-
Vaginal	224	88	39.3						
Cesarean section	191	64	33.5						
*Perceived newborn’s risk*									
Yes	218	79	43.9	0.049	1.18	1.00–1.39	-	-	-
No	197	60	33.7						

*Notes*: HS: Health service. PR: Prevalence ratio. CI: Confidence interval.

*P value* significant ≤ 0.05. Minimum monthly salary = R$ 998.00.

Completion of the five tests was more prevalent for the newborns whose mothers had the following characteristics: primiparous, older, better educated, married or living in a stable union, income less than or equal to minimum monthly salary, beneficiaries of the *Bolsa Familia* (Family Grant) program, residents in the state capital, and considered the neonatal period to be risky to the health of newborns. We also found a higher prevalence of complete NS tests in cases in which the mothers were offered additional or better care, such as guidance on the tests and searches for post-discharge health services (Table 2).

After we included the variables of individual characteristics (maternal and care) with *p* ≤ 0.20 in the multivariate model (Poisson regression), the only variables that remained associated with the outcome were living in the state capital (PR = 1.36, CI = 1.18–1.56) and receiving guidance from health professionals on the five NS tests during hospitalization (PR = 1.30, CI = 1.08–1.67), with an estimated prevalence of 80.6% for both conditions (Table 2).

The highest prevalence in the bivariate analysis of the institutional characteristics for completing the five tests (*p* ≤ 0.001) was associated with municipal administration, low complexity, and a lower ratio of professionals per hospital bed (Table 3). Slightly more of the newborn subjects were male (52,5%); their average gestational age was 39.2 weeks; and their average birth weight was 3.31 kg. None of the newborns’ characteristics showed any association with completion rates for the five tests (Table 3).

**Table 3:** Bivariate analysis of institutional and newborn characteristics with the of the five NS tests (n = 152). Natal/Brazil, 2019.

		Neonatal screening tests			*p value*
	n(415)	n	%	CI	
**Care characteristics**					
*Administration*					**<0.001**
Municipal	190	88	46.3	39.2–53.4	
State	96	11	11.5	5.1–17.8	
Federal	129	53	41.1	32.2–49.6	
*Level of complexity*					
Low risk	190	88	46.3	39.2–53.4	**<0.001**
High risk	225	64	28.4	22.5–34.3	
*Ratio between professionals/beds*					
≤ 0.7	198	92	42.5	39.5–53.4	**<0.001**
> 0.7	217	60	27.6	21.7–36.6	
**Newborn characteristics**					
*Sex*	218	79	36.2	29.9–42.6	0.863
Male	197	73	37.1	30.3–43.2	
Female					
	**Md (AIQ 25–75)**				***p value***
*Gestational age*	39.2 (38.4–40.1)				0.097 ţ
*Weight at birth*	3.310 (3.010–3.620)				0.165 ţ
*Length at birth*	49.0 (48–50)				0.049 ţ
*Head circumference at birth*	35.0 (34–36)				0.713 ţ

*Notes*: * Ratio between the number of professionals per obstetric beds in the rooming-in unit. Md: Median. AIQ: Interquartile range. Weight in kilograms, length, and head circumference in centimeters. CI: Confidence interval. Pearson’s chi-squared test.

ţ Mann-Whitney test. *P value* significant at ≤ 0.05.

## Discussion

The *PNAISC* reaffirms the rights of children guaranteed by the 1988 Constitution and the Statute for Children and Adolescents. Comprehensive and integrated care must be a priority to reduce infant morbidity and mortality, especially in the neonatal period, in which the mortality rate corresponds to 70% of the infant mortality rate. Universal NS is an essential part of the humanized and qualified care for newborns, and must be performed in a timely manner for early diagnosis and intervention, bringing greater benefits, social inclusion, and enhancing the quality of life for children affected by any of the identified pathologies [3].

Despite the law having mandated their completion within the first 48 hours of a newborn’s life, we found less than 100% compliance in the maternity wards for completing, the pulse oximetry screening, red reflex examination, hearing screening, and tongue tests, which probably reflects the national reality [11,14,15]. The likely reasons for this are institutional deficiencies (e.g., inadequate training, resources, or instructions; failure to enforce the law, etc.), and healthcare personnel’s lack of understanding of the tests’ critical role in newborns’ health. It might be that the mandatory *TNU* policies are in place but not being followed or enforced because the maternity wards lack sufficient numbers of properly trained personnel, testing equipment or lab services, or time to provide the care required for mother and child.

There were some variations in the data collection points. While there was a low prevalence for completing the red reflex examination in the first 48 hours, the prevalence increased by 37.6% after the newborns’ discharge from the hospital or at 28 days of life. One institution reported lacking the ophthalmoscope necessary to identify the beam of light during the data collection period, which almost certainly contributed to the test not being done for some of the children in this study. An ophthalmologist performed the procedure in only one of the maternities while in the others it was performed by pediatricians.

We found a higher prevalence for the Guthrie test because it has been instituted and required since 1976, but the prevalence rate was only 63.6%, and the test was done between the newborns’ third and seventh days. This rate was lower than the national prevalence rate (70.8%) found in the National Health Survey (*PNS* per its Portuguese acronym) performed in Brazil in 2013, and low compared with other countries in Latin America (99%) [16], even though the prevalence rate in rural settings increased by approximately 10% between 2004 and 2017 [10] The *PNS* shows regional inequalities, with a higher prevalence of testing in the South and Southeast regions. When we analyzed the results at the end of the neonatal study period, we found considerable improvement over the prevalence reported in the Neonatal Call Survey conducted in the state of RN in 2010 [14], but lower than that of the 2013 NHS survey [10]. Therefore, considering that early testing is essential for the newborn’s health, this result was not significant.

For the most accurate diagnosis of the studied pathologies, the recommended period of biological screening for universally attending all newborns is between the third and the fifth days of life, when there is a more significant accumulation of metabolites in the blood [3]. The children are typically already at home by this period, so the test should be carried out at a primary health unit; attendance at the maternity hospital is only mandatory for a hospitalized newborn. However, technical, structural, organizational, access, and post-partum issues can compromise the test’s expected prevalence rate, leading to a failure to comply with the country’s current legislation and polices. This testing period differs from those recommended by developed other countries. For example, Italy’s, Ministry of Health recommends that newborns’ biological screening be performed between 48 to 72 hours after the birth [17]. This earlier test date could hasten the diagnosis, initial treatment, and medical follow-up of some diseases. Therefore, we recommend that these screenings be conducted soon as possible after the 48 hours point, under appropriate clinical conditions for the newborns and mothers in order, to obtain the most accurate results. We believed that early screening (i.e. the Guthrie test) and data collection in maternity hospitals could improve infant morbidity and mortality.

The lowest prevalence rates among the five tests were for the hearing and tongue tests. One reason for this might be the low numbers of speech therapists in maternity hospitals (and the negligible number in primary healthcare units). These tests need to be performed during the hospitalization, since so many patients live in rural areas without easy access to specialists, and an adverse result for these tests can signal imminent issues with feeding and boding. The lack of equipment, qualified human resources, and protocol options are known barriers to these two tests being performed early [15].

The coverage for performing the hearing screening test found in this study is higher than that found in the literature. While Paschoal et al. [11] identified a national performance rate of up to 37.2% for 2008–2015, Mallmann et al. [10] identified a performance rate of 44.1% in 2013 for children of any age in northeastern Brazil. The results for Brazil are lower than those presented by Bouillot et al. [18] for France in 2016 (99.7%). Despite the legal obligation to conduct a hearing test on every newborn in the first 24 to 48 hours of life, the Multidisciplinary Committee on Hearing Health relaxes the standards to 95% compliance and a deadline of the end of the first month with the evaluation broken into two stages (test and retest) [19].

The tongue test to identify ankyloglossia, a genetic disorder which affects approximately 11% of newborns [20] and can compromise breastfeeding and speech, had the lowest prevalence in the present study, and received less attention in the guidance provided by the multidisciplinary team. Despite being mandated by law since 2014 to be carried out by trained professionals and through a specific protocol [21], public actions have been minimal. The *PNAISC* rarely references this test and says that it should only be performed when necessary [3]. The Brazilian Society of Pediatrics advises against performing the test at all, alleging that because of the low incidence and the lack of scientific evidence in the protocols, it should be a routine examination in pediatric practice rather than a newborn test [22]. However, the literature reveals adequate protocols, such as the Bristol protocol, guided by the Brazilian Ministry of Health. We need to rectify this lack of understanding among health professionals and pregnant women of the test’s importance for breastfeeding [7,23].

The *PNTN* in the state of Rio Grande do Norte is in the process of qualifying two kinds of teams: teams comprising speech therapists, pediatricians and nurses to identify ankyloglossia, and teams comprising pediatric surgeons and dental surgeons to perform the frenectomies. The follow-up and collection phases will start after this multiplier training phase. Thus, better compliance is expected in the coming years.

Pulse oximetry screening and the Guthrie test are generally performed by nursing technicians, who are found in greater numbers in maternity hospitals. However, the lower rates for performing the Guthrie test could also be due to the small number of trained professionals available at any given time because of vacations, leaves, or cutbacks. This leads to the test being performed in primary health care, where the difficulties are even greater. These practitioners often have smaller staffs, leading them to limit their testing days and hours; they also might be less likely to explain the importance of these tests to pregnant women. However, active Family Health Strategy teams that strengthen the bond between professionals and families and facilitate access and follow-up can produce better results [24]. Thus, we need to improve the interactions between basic and specialized care to ensure that these tests are conducted promptly [25].

In addition to regional inequities, Brazil’s current economic and political situation especially with the added stressors of the COVID-19 pandemic has also reduced the resources available for primary healthcare. The Family Health Strategy forecast until 2030 is for less coverage and an increase in morbidity and mortality [26]. This will likely compromise the *PNTN’*s collection, analysis, communication and follow-up after diagnosis.

Our findings did not differ significantly from prior research, which also pointed to inequities based on regional, income, and educational differences [10,11]. Rural residents’ difficulty in accessing adequate healthcare is a national reality characteristic of developing countries [10]. Better education can also lead to greater awareness among pregnant women and mothers of the importance and availability of newborn testing and healthcare services. Finally, people with higher incomes often have more healthcare knowledge and understand the importance of supplementing or replacing home based healthcare with care from primary health units or private medical offices for the examinations.

One variable that remained in the final model of the proposed regression was the provision of guidance on the NS test. The *PNAISC* and the *PNTN* legislation both recommend that this occurs in the maternity ward and during the post-partum period, as some tests can be done, preferably, until the end of the first month. In this sample, only a third of mothers received guidance on the importance of TNU in the maternity ward, where most tests were performed without any explanation. Most of the mothers knew something about the tests from their providers’ prenatal care, other resources (e.g., Internet, library, friends or family, etc.), or previous childbearing experience. However, most did not fully understand the purpose, importance, recommended test guidelines period, or the diseases screened [27]. Higher levels of this knowledge are often associated with higher education and maternal income levels, but the absence of this information could reflect flaws in the healthcare services. The first guidelines should ideally be given during prenatal care to allow time for assimilation, especially since mothers might be more worried, anxious, or tired than usual during the first 48 hours after giving birth [23,28,29]. The dedication, cooperation, and appreciation of the team, and more comprehensive professional training can contribute to better results [8].

The literature suggests that high-risk maternities have better structure and involve more qualified professionals, equipment, supplies, and best practices that can improve the quality of care during childbirth and birth [30]. Nevertheless, more infants in our study were screened in low-complexity maternity hospitals with fewer professionals per bed.

Brazil’s high-risk maternity hospitals which are part of the Healthcare Network are primarily located in major municipalities. They also received low-risk pregnant women during our research period, which increased the number of patients relying on the same resources. Excessive demand for the most complex services could interfere with the quality of overall care. However, having a larger number of professionals per bed in the rooming-in unit did not prove to be an essential factor in our study. This reveals that the responsibility for providing care can overcome the most common challenges All the institutions we surveyed also conducted teaching activities and continuous team training, which could have enabled better care.

The added pressures of the COVID-19 pandemic, might lead to even lower rates of newborn testing. Due to officially imposed and voluntary stay-at-home measures, people have abstained from all but the most critical healthcare interactions to avoid coming into contact with the virus. The need to focus on COVID-19 patients has meant that many healthcare providers have reduced some health services or shifted to telehealth to respond to the current emergency. Others have had to deal with their own infections or those in their families. Research suggests that in addition to the increase in mortality due to the virus, the pandemic will also indirectly increase mortality. Roberton et al. [31] estimated a possible increase in the mortality rates of mothers and children aged 0–5 caused by interruption in healthcare systems and coverage, decreased access to services, and less food, all of which were common in previous epidemics. The forecast of a decrease in the supply of services and an increase in mortality resulting from the scarcity of resources will be exacerbated by the COVID-19 pandemic, with grave results for newborns and mothers.

### Study limitation

This study has some limitations, such as having been carried out only in the state capital. However, the study likely not only reflects the local reality but the country’s reality. The study revealed inequities which, are likely to be accentuated in rural maternity hospitals, because they generally have fewer resources. However, the study’s strengths include being a longitudinal study that engaged with the mothers from the maternity ward to the end of the neonatal period, thus avoiding the memory biases common in extremely long-term investigations. Furthermore, this study contributed new findings on the still relatively understudied topic of universal NS, adding valuable insights, particularly on pulse oximetry screening, the red reflex examination and the tongue test.

## Conclusions

None of the NS tests met the full coverage within the ideal testing period recommended by the policies. The reasons included institutional, social and welfare factors. Achieving optimal coverage will always be challenging. However, this study’s, findings suggest that improvements could be realized by restructuring various institutions, regulating the care network, adapting processes, expanding the number of qualified professionals and promoting family involvement. It is important to longitudinally prioritize the educational actions, service quality care and public policies, and social conditions that impact the health services of the population most in need of SUS.
